# Molecular Traces of Gastric Cancer in Saliva: From Tissue Signatures to Salivary *SLC5A5* as a Potential Biomarker

**DOI:** 10.1002/ueg2.70221

**Published:** 2026-05-23

**Authors:** Catarina Lopes, Andreia Brandão, Dimitris Vavoulis, Sofia Paulino, João Costa, Inês Marques de Sá, Sara Archer, Ricardo Küttner‐Magalhães, Ricardo Marcos‐Pinto, Diogo Libânio, Mário Dinis‐Ribeiro, Carina Pereira

**Affiliations:** ^1^ Precancerous Lesions and Early Cancer Management Group Research Center of IPO Porto (CI‐IPOP)/CI‐IPOP@RISE (Health Research Group) Portuguese Oncology Institute of Porto (IPO Porto)/Porto Comprehensive Cancer Center Raquel Seruca (Porto.CCC) Porto Portugal; ^2^ Center for Health Technology and Services Research (CINTESIS@RISE) University of Porto Porto Portugal; ^3^ ICBAS—School of Medicine and Biomedical Sciences University of Porto Porto Portugal; ^4^ Cancer Genetics Group Research Center of IPO Porto (CI‐IPOP)/CI‐IPOP@RISE (Health Research Group) Portuguese Oncology Institute of Porto (IPO Porto)/Porto Comprehensive Cancer Center Raquel Seruca (Porto.CCC) Porto Portugal; ^5^ Department of Oncology Oxford Molecular Diagnostic Center University of Oxford Oxford UK; ^6^ Biomedical Research Center The Wellcome Center for Human Genetics University of Oxford Oxford UK; ^7^ Department of Pathology Portuguese Oncology Institute of Porto Porto Portugal; ^8^ Department of Gastroenterology Portuguese Oncology Institute of Porto Porto Portugal; ^9^ Centro Hospitalar Universitário de Santo António Porto Portugal

**Keywords:** biomarkers, gastric lesions, liquid biopsy, personalized medicine

## Abstract

**Background:**

Early detection of gastric cancer (GC) and reliable risk stratification for metachronous gastric lesions (MGLs) remain societal and clinical challenges, particularly in intermediate risk populations. Non‐invasive approaches such as saliva‐based biomarkers could complement current strategies. The aim of this study was to identify and validate a tissue‐based gene expression signature for early gastric lesions, explore its potential for MGL prediction, and assess its detectability in saliva.

**Methods:**

Three studies were conducted: (1) a retrospective case control study to identify (RNA sequencing with machine learning) and validate (reverse transcription [RT] quantitative polymerase chain reaction [qPCR]) a gene expression signature for early gastric cancer using formalin‐fixed paraffin‐embedded (FFPE) samples, (2) a retrospective longitudinal study evaluating the ability of the signature to stratify MGL risk, and (3) a prospective study testing the signature in saliva using droplet digital (dd)PCR in patients with gastric lesions and endoscopy‐confirmed controls.

**Results:**

A six‐gene tissue‐based expression signature (*ADAMTSL1, CCNA2, HSP90AB1, HSPD1, PSAPL1, and SLC5A5*) robustly discriminated early gastric lesions from non‐tumor mucosa (area under the curve (AUC) = 0.96 and 95% confidence interval [CI]: 0.94–0.99). Models tailored for MGL prediction, incorporating clinical variables, achieved moderate performance (AUC = 0.74 and 95% CI: 0.59–0.88). In saliva, only the *SLC5A5* gene showed consistent dysregulation. When combined with age and sex, the model reached an AUC of 0.78 (95% CI: 0.69–0.88) for the non‐invasive detection of early GC, with a positive predictive value of 0.69 and negative predictive value of 0.81.

**Conclusion:**

This study presents a validated tissue‐based gene signature for early GC detection and exploratory MGL risk stratification. Salivary *SLC5A5* shows potential as a non‐invasive biomarker, though its utility requires further validation in dedicated saliva‐based studies.

## Key Summary

- Summarize the established knowledge on this subject.
  ◦ Early gastric cancer benefits from endoscopic detection, but current screening and surveillance strategies are invasive and resource intensive.
  ◦ Reliable non‐invasive biomarkers for early gastric lesions and metachronous gastric lesion risk stratification are lacking.
  ◦ Tissue‐based molecular signatures have shown diagnostic potential but limited translational application.
  ◦ Saliva is an emerging biofluid for cancer biomarker discovery, though evidence in gastric cancer remains scarce.
- What are the significant and/or new findings of this study?
  ◦ A six‐gene tissue expression signature accurately discriminated early gastric lesions from non‐tumor mucosa.
  ◦ The signature showed moderate ability to stratify the risk of metachronous gastric lesions when combined with clinical variables.
  ◦ Among the six genes, only *SLC5A5* showed consistent dysregulation in saliva.
  ◦ A salivary model incorporating *SLC5A5*, age, and sex achieved modest accuracy for non‐invasive detection of early gastric cancer.

## Graphical abstract

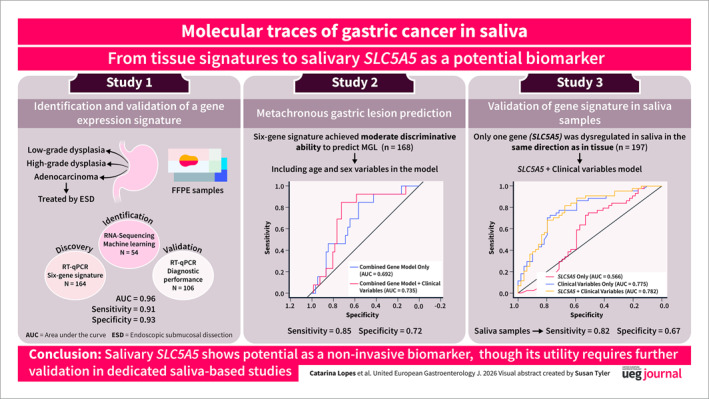

**Abbreviations**
AUC: Area under the ROC curve
cDNA: Complementary DNA
CI: Confidence interval
DOR: Diagnostic odds ratio
ESD: Endoscopic submucosal dissection
FC: Fold change
FFPE: Formalin‐fixed paraffin‐embedded
GC: Gastric cancer
GI: Gastrointestinal e
GTEx: Genotype‐tissue expression
MGL: Metachronous gastric lesion
NAT: Normal adjacent tissue
NPV: Negative predictive value
OR: Odds ratio
PCA: Principal component analysis
PPV: Positive predictive value
ROC: Receiver operating characteristic
TCGA: The Cancer Genome Atlas

## 1 Introduction

Gastric cancer (GC) remains a pressing global health issue, ranking among the leading causes of cancer‐related mortality worldwide [1]. Prognosis improves markedly when detected early, yet most patients in intermediate‐risk regions continue to present with advanced stage [2, 3]. Unlike high‐incidence countries such as Japan and South Korea, where structured screening programs enable systematic detection of early disease, Western settings lack standardized approaches despite a non‐negligible disease burden [4, 5, 6]. Consequently, there are frequent late‐stage diagnoses, highlighting the need for improved, accessible, cost‐effective, and preferably non‐invasive tools for early detection.

Upper gastrointestinal (GI) endoscopy is the diagnostic gold standard, offering high sensitivity for early lesions [7]. However, its invasiveness, cost, and limited scalability make it impractical for population‐wide screening, especially in intermediate‐risk settings [8, 9]. Molecular markers could serve as complementary tools to refine risk stratification and guide surveillance more efficiently.

Transcriptomic profiling has opened new avenues for biomarker discovery in GI oncology, holding promise for distinguishing precancerous and early‐stage gastric lesions from non‐malignant mucosa [10]. Yet, most remain unvalidated and lack clinical translation [11, 12, 13]. Beyond diagnosis, clinical management also requires effective surveillance: 10%–20% of patients develop metachronous gastric lesions (MGLs) after resection of early GC or precancerous conditions [14]. This risk is particularly relevant after endoscopic submucosal dissection (ESD), where gastric preservation is prioritized [15]. Current surveillance relies solely on annual endoscopy, a “one‐size‐fits‐all” strategy which ignores patient‐specific biological heterogeneity and may lead to both over‐ and under‐surveillance [16, 17].

Considering this landscape, we aimed to identify robust transcriptomic alterations associated with early gastric neoplasia and evaluate their relevance across different clinical contexts. Our objectives were: (1) to define and validate a tissue‐based gene expression panel associated with early gastric lesions, (2) to examine whether this panel contains signals relevant to future MGL development, and (3) to explore whether selected tissue‐derived targets are detectable in a completely non‐invasive matrix.

Emerging evidence shows that tumors can release nucleic acids, proteins, and extracellular vesicles into multiple body fluids, including saliva [18]. Although the stomach is anatomically distant from the oral cavity, gastric epithelial cells and tumor‐derived vesicles can enter systemic circulation and reach salivary glands, where molecular cargo may be secreted into saliva [19]. Several cancers, such as esophageal [20], liver [21], and lung [22], have demonstrated detectable biomarkers in saliva, indicating that distal malignancies can generate measurable salivary molecular signatures. This supports the exploratory assessment of whether gastric mucosal transcriptomic alterations leave detectable traces in saliva.

We developed a multi‐stage approach, beginning with RNA‐sequencing of formalin‐fixed paraffin‐embedded (FFPE) samples spanning normal mucosa, dysplasia and early adenocarcinoma, followed by targeted validation in an expanded FFPE cohort. We then examined whether the panel was associated with the risk of MGL development after ESD. Finally, we examined the detectability and diagnostic behavior of selected transcripts in saliva, aiming to determine whether any molecular signals originating in gastric tissue meaningfully extended to a fully non‐invasive sampling matrix.

Taken together, these analyses provide a structured assessment of early GC–associated molecular changes, combining robust tissue‐based discovery with exploratory extensions toward risk stratification and non‐invasive sampling. Although the latter applications remain preliminary, they highlight which components of the signature show biological consistency across contexts and may serve as a foundation for future, dedicated biomarker development.

## 2 Methods

### 2.1 Study Design and Patient Cohort

This work comprises three complementary studies conducted to identify and validate early GC biomarkers using tissue and saliva samples. All studies were approved by the Ethics Committee of IPO Porto (CES IPO: 100/024) and conducted according to the Declaration of Helsinki. Written informed consent was obtained from all participants. This study followed the STROBE guidelines for observational studies.

#### 2.1.1 Retrospective Case‐Control Study for Tissue Biomarker Discovery and Validation (Study 1)

FFPE tissue samples were consecutively retrieved from the histopathology archives and the ESD‐treated cohort of patients based on sample availability and clinical follow‐up. Controls included histologically normal mucosa (non‐tumor) from GC patients who underwent gastrectomy between January 2014 and December 2015 (*n* = 130). All control samples were reviewed by a pathologist and confirmed to have no chronic inflammation. Sample locations (antrum vs. corpus) were recorded and are summarized in Table 1. Cases included low‐grade dysplasia (LGD), high‐grade dysplasia (HGD), or adenocarcinoma (ADC) samples from patients treated by ESD between January 2012 and September 2018 (*n* = 168). Some individuals contributed multiple samples, yielding 337 samples from 298 participants.

**TABLE 1:** Clinicopathological features of patients included in the tissue‐based study.

*N* participants/samples	Identification phase *N* = 51/54		Discovery phase *N* = 164/167		Validation phase *N* = 106/112	
	Controls	Cases	Controls	Cases	Controls	Cases
	*N* = 23/26	*N* = 28/28	*N* = 66/69	*N* = 96/98	*N* = 48/54	*N* = 56/58
Age, years						
Mean ± SD	71.2 ± 9.2	72.0 ± 7.9	70.0 ± 10.6	68.9 ± 9.2	67.9 ± 9.9	67.5 ± 9.8
Median (Min‐Max)	72 (54–85)	71 (58–85)	70.5 (38–88)	69 (51–90)	67.5 (49–88)	68 (45–85)
Sex, *n* (%)						
Female	10 (43.5)	8 (28.6)	23 (34.8)	42 (42.9)	15 (31.3)	18 (32.1)
Male	13 (56.5)	20 (71.4)	43 (65.2)	56 (57.1)	33 (68.7)	38 (67.9)
HP status, *n* (%)						
Positive	**2 (8.7)**	**2 (7.1)**	**6 (9.1)**	**8 (8.2)**	**0 (0.0)**	**6 (10.7)**
Negative^a^	**5 (21.7)**	**22 (78. 6)**	**5 (7.6)**	**77 (78.5)**	**4 (8.3)**	**44 (78.6)**
Unknown	**16 (69.6)**	**4 (14.3)**	**55 (83.3)**	**13 (13.3)**	**44 (91.7)**	**6 (10.7)**
Localization^b^, *n* (%)						
Antrum	11 (42.3)	17 (60.7)	19 (27.5)	67 (68.3)	17 (31.5)	41 (70.7)
Cardia	0 (0)	0 (0)	0 (0)	3 (3.1)	0 (0)	1 (1.7)
Corpus	15 (57.7)	11 (39.3)	50 (72.5)	14 (14.3)	36 (66.7)	5 (8.6)
Incisura angularis	0 (0)	0 (0)	0 (0)	10 (10.2)	0 (0)	8 (13.8)
Stump	0 (0)	0 (0)	0 (0)	0 (0)	0 (0)	2 (3.5)
Corpus‐antrum transition	0 (0)	0 (0)	0 (0)	4 (4.1)	1 (1.8)	1 (1.7)
Histology, *n* (%)						
LGD		4 (14.3)		20 (20.4)		10 (17.2)
HGD		8 (28.6)		42 (42.9)		21 (36.2)
ADC		16 (57.1)		36 (36.7)		27 (46.6)
MGL development, *n* (%)						
Yes		12 (42.9)		25 (25.5)		14 (24.1)
No		16 (57.1)		73 (74.5)		44 (75.9)

Abbreviations: ADC, adenocarcinoma; FFPE, formalin‐fixed paraffin‐embedded; HGD, high‐grade dysplasia; HP, *Helicobacter pylori*; LGD, low‐grade dysplasia; MGL, metachronous gastric lesion; SD, standard deviation.

^a^ Erradicated HP cases were classified as negative.

^b^ Normal mucosa in controls, lesion localization in cases. Values in **bold** are statistically different between groups (regardless of set).

Three analytical phases were conducted:

1. Identification of an early GC gene signature (*n* = 51, identification phase);
2. Construction of a predictive model (*n* = 164, discovery phase);
3. Validation in an independent set (*n* = 106, validation phase).

#### 2.1.2 Retrospective Cohort Study to Assess Metachronous Gastric Lesion Prediction (Study 2)

This study builds upon the case group from Study 1. Patients with superficial gastric lesions who underwent ESD and had ≥ 3 years of follow‐up were stratified into MGL (*n* = 39) and No MGL (*n* = 129). MGLs were defined as new lesions detected during follow‐up at an anatomical site distinct from the index lesion.

#### 2.1.3 Prospective Saliva‐Based Biomarker Validation (Study 3)

Unstimulated whole saliva samples were prospectively and consecutively collected between October 2021 and February 2025 from 95 early GC patients and 102 endoscopy‐confirmed controls at IPO‐Porto, *Centro Hospitalar Universitário de Santo António* (CHUdSA), and *Invicta Saúde—Clínica Central da Areosa*. Controls were individuals undergoing upper GI endoscopy for routine or screening purposes, without histologically confirmed gastric lesions. Most were asymptomatic or referred for non‐specific GI complaints, namely dyspepsia. Saliva was collected before ESD (cases) or endoscopy (controls) under fasting conditions, aiming to minimize variability due to dietary intake and circadian fluctuations in salivary composition. Approximately 1–4 mL of unstimulated whole saliva was collected per participant. Samples were centrifuged at 2600 x g for 15 min at 4°C, treated with SUPERase•InTM RNase Inhibitor (Invitrogen, Waltham, MA, USA), and stored at −80°C until RNA extraction. Saliva processing aimed to minimize RNA degradation, recognizing the inherent instability of free mRNA in this matrix. Four participants provided two samples at different time points, due to the diagnosis of a subsequent lesion.

### 2.2 RNA Extraction

RNA was extracted from FFPE sections using the RNeasy FFPE Kit (Qiagen, Hilden Germany) with on‐column DNase I treatment (Qiagen, Hilden, Germany) and from saliva using the miRNeasy Mini Kit (Qiagen, Hilden, Germany), following the manufacturer's guidelines. All RNA samples were stored at −80°C until further analysis.

### 2.3 RNA‐Sequencing and Gene Expression Panel Selection

RNA‐seq was conducted on 28 early GC and 26 non‐tumor FFPE samples (DV200 > 30%) using Illumina NovaSeq 6000 after rRNA depletion. Reads were quantified with Salmon (version 1.4.0) and differential expression analysis was carried out using DESeq2 (|log2FC| > 2, adjusted *p* < 0.05) [23].

Feature selection and model construction employed random forest (RF), gradient boosting machine (GBM), and DT algorithms using the tidymodels framework [24]. The analytical pipeline included data preprocessing, training/test splitting, feature selection, and model tuning using cross‐validation. A detailed description of preprocessing steps, resampling strategy, feature selection procedures, and hyperparameter tuning is provided in Supporting Information S1. Details on the hyperparameter grids and tuning ranges used for RF, GBM, and DT models are provided in Supporting Information S1: Table S1. The identified gene expression signature was corroborated using publicly available data on The Cancer Genome Atlas (TCGA—stomach adenocarcinoma [STAD] cohort) and Genotype‐Tissue Expression (GTEx) portal, filtering for stage I GC patients (81 stage I GC, 7 matched normal adjacent tissue (NAT), 174 normal samples). The clinicopathological characteristics of the included GC cases from the TCGA‐STAD cohort are summarized in Supporting Information S1: Table S2.

### 2.4 Reverse Transcription Reaction

Complementary DNA (cDNA) was synthesized from up to 1 μg of RNA from the entire cohort of FFPE samples using the High‐Capacity cDNA Reverse Transcription Kit (Applied Biosystems, Waltham, MA, USA), according to the manufacturer's protocol. To assess RT reaction quality, 1 μg of QPCR Human Reference Total RNA (Agilent, Santa Clara, CA, USA) was included in every run. Genomic DNA (gDNA) contamination was evaluated by processing four random samples with and without reverse transcriptase enzymes. The gDNA contamination was approximately 0.1%, deemed negligible.

### 2.5 Real‐Time PCR

Real‐time quantitative PCR (qPCR) was performed using the QuantStudio 5 Real‐Time PCR System (Applied Biosystems, Waltham, MA, USA) in multiplex panels. Each multiplex panel included four genes, as summarized in Supporting Information S1: Table S3.

The most suitable reference gene was chosen based on the most stable gene identified in RNA‐seq analysis (*TADA2B*). This gene exhibited expression levels comparable to those of the selected target genes and had the lowest coefficient of variation (CV) value (0.0689, Supporting Information S1: Table S4).

Each mRNA quantification was performed in triplicate. Missing C_*T*_ values were treated as gene downregulation, and the highest C_*T*_ for that specific gene plus 1 was used as a placeholder.

### 2.6 Droplet Digital PCR

Gene expression in saliva was assessed by ddPCR using the QX600 AutoDG Droplet Digital PCR System (Bio‐Rad, Hercules, CA, USA). Reactions were prepared with the One‐Step RT‐ddPCR Advanced Kit for Probes (Bio‐Rad, Hercules, CA, USA) in multiplex panels. The multiplex panel composition is detailed in Supporting Information S1: Table S5. Gene expression was normalized to reference genes (ratio between the concentration (copies/μL) of the target gene and the mean concentration of the reference genes), log‐transformed, and outliers removed prior to analyses.

### 2.7 Data Processing and Statistical Analysis

All data processing and analyses were performed in RStudio (version 2024.12.1) using R software (version 4.4.3). A list of packages used and corresponding versions is available in Supporting Information S1: Table S6. Missing or unknown data were excluded from statistical analyses on a per‐variable basis, and analyses were conducted using available‐case data. Continuous variables were compared using t‐test or Wilcoxon test and categorical variables using χ2 or Fisher's exact test. To adjust for multiple comparisons, false discovery rate (FDR) method was employed, with a significance threshold set at *p* < 0.05.

Receiver operating characteristic (ROC) curves, logistic regression, and area under the ROC curves (AUC) were used to assess predictive performance, including multivariable models with clinical variables, namely age and sex. For each multivariate model, the complete logistic regression formula is as follows, with gene expression values standardized (z‐score transformed) and clinical covariates (age, sex) included:

$$\text{logit}\left(P\right)=\beta_{0}+\beta_{1}\times ADAMTSL1+\beta_{2}\times CCNA2+\beta_{3}\times HSP90AB1+\beta_{4}\times HSPD1+\beta_{5}\times PSAPL1+\beta_{6}\times SLC5A5+\gamma_{1}\times \text{Age}+\gamma_{2}\times \text{Sex}$$

where $\beta_{0}$ is the intercept, $\beta_{1}...\beta_{6}$ are gene coefficients, and $\gamma_{1},\gamma_{2}$ are coefficients for clinical covariates. Depending on the specific analysis, models were constructed using genes only, clinical covariates only, or a combination of both. The estimated coefficients for all models are reported in Supporting Information S1: Table S7. Because the objective was discrimination at a fixed minimum 3‐year follow‐up and censoring was negligible, MGL development was modeled as a binary outcome. ROC‐based metrics were therefore used to evaluate predictive performance.

Sensitivity, specificity, positive predictive value (PPV), negative predictive value (NPV), and additional metrics (positive and negative likelihood ratios [LR+ and LR−, respectively] and diagnostic odds ratio [DOR]) were calculated. Model calibration was assessed using the Brier score. Further methodological details are provided in the Supporting Information S1.

## 3 Results

### 3.1 Gene Expression Profiling Reveals Candidate Biomarkers for Early Gastric Cancer Detection (Study 1—Identification Phase)

Clinicopathological characteristics of participants across the tissue‐based study phases are summarized in Table 1. The number of samples might differ from the number of participants, and accordingly, some variables refer to participants while others refer to samples. Across phases, males predominated (57%–71%, *p* > 0.05) and mean ages were similar between cases and controls (68–72 years, *p* > 0.05). Gastric lesions were mostly located in the antrum (61%–71%, *p* > 0.05) and adenocarcinoma was the most frequent histology in the identification and validation phases (*p* > 0.05). *Helicobacter pylori* (HP) infection status was unknown for a subset of control samples due to the retrospective nature of gastrectomy tissue collection. MGL frequency ranged from 43% (identification) to 24% (validation). The total follow‐up time was 129 months.

**FIGURE 1:**
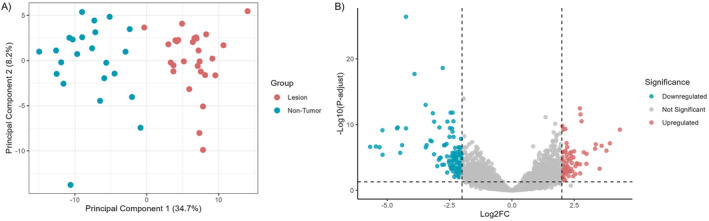
Clustering and differential expression of genes in gastric lesions. (A) Principal component analysis (PCA) showing distinct clustering patterns between gastric lesion and normal mucosa samples, highlighting the separation of the two groups. (B) Volcano plot of the differential expression analysis showing 180 genes with |log2FC| > 2 and P‐adjust < 0.05. Of these, 75 genes were upregulated (red) and 105 were downregulated (blue). FC: fold change.

RNA‐seq differential expression analysis in the identification set (28 cases, 26 controls) revealed 180 differentially expressed genes (DEGs, |log2FC| > 2, P‐adjust < 0.05), with clear case‐control clustering in the principal component analysis (PCA, Figure 1A). Of these, 75 were upregulated and 105 were downregulated in gastric lesions (Figure 1B).

**FIGURE 2:**
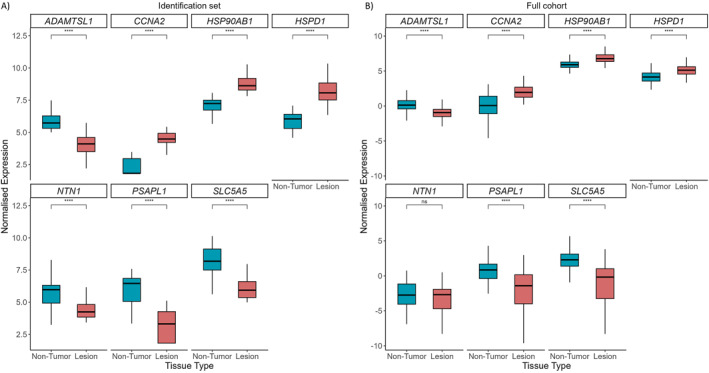
Gene expression profile of selected biomarkers in the identification set (A) and full cohort (B). Boxplots show the expression levels of the seven genes identified as potential biomarkers for lesions. *CCNA2, HSP90AB1* and *HSPD1* exhibit significantly higher expression in lesion samples, while *ADAMTSL1, NTN1* (only in the identification set), *PSAPL1*, and *SLC5A5* show lower expression. ****p < 0.0001.

Feature selection identified a seven‐gene panel (*ADAMTSL1, CCNA2, HSP90AB1, HSPD1, NTN1, PSAPL1* and *SLC5A5*, Supporting Information S1: Figure S1), with distinct expression profiles (Figure 2A). Upregulated genes included *CCNA2* (FC = 2.88, *P* = 1.6 × 10^−6^), *HSP90AB1* (log2FC = 2.02, *P* = 1.6 × 10^−10^), and *HSPD1* (log2FC = 2.72, *P* = 3.3 × 10^−13^), while *ADAMTSL1* (log2FC = −2.10, *P* = 1.1 × 10^−6^)*, NTN1* (log2FC = −2.14, *P* = 2.7 × 10^−7^)*, PSAPL1* (log2FC = −3.2, *P* = 3.4 × 10^−8^), and *SLC5A5* (log2FC = −2.46, *P* = 1.5 × 10^−12^) were downregulated compared to non‐tumor mucosa.

### 3.2 The Gene Expression Signature Exhibits Consistent Dysregulation in Publicly Available Datasets (Study 1—In Silico Validation)

To validate this seven‐gene panel, stage I GC samples from TCGA‐STAD and GTEx were analyzed. NAT samples from TCGA and non‐tumor tissues from GTEx were combined due to their overlap in PCA (Supporting Information S1: Figure S2). All genes showed consistent dysregulation patterns with our dataset (Supporting Information S1: Figure S3): *CCNA2* (log2FC = 3.73, *p* = 6.13 × 10^−33^), *HSP90AB1* (log2FC = 1.20, *p* = 1.40 × 10^−20^) and *HSPD1* (log2FC = 1.31, *p* = 2.28 × 10^−19^) were upregulated in stage I GC, while *ADAMTSL1* (log2FC = −1.37, *p* = 1.17 × 10^−14^), *NTN1* (log2FC = −1.95, *p* = 5.57 × 10^−15^), *PSAPL1* (log2FC = −3.32, *p* = 1.96 × 10^−9^), and *SLC5A5* (log2FC = −3.03, *p* = 6.30 × 10^−8^) were downregulated.

### 3.3 Targeted Gene Expression Analysis Confirms Differential Expression and Diagnostic Potential of Candidate Biomarkers in the Discovery Cohort (Study 1—Discovery Phase)

All seven candidate genes were measured by RT‐qPCR in the extended cohort (*n* = 337 samples), with six showing significant differential expression between early GC and controls (*p* < 0.05, Figure 2B). *CCNA2* (log2FC = 4.24, *p* < 0.0001)*, HSP90AB1* (log2FC = 1.89, *p* < 0.0001), and *HSPD1* (log2FC = 2.00, *p* < 0.0001) were markedly upregulated in lesion samples, whereas *ADAMTSL1* (log2FC = −2.25, *p* < 0.0001)*, PSAPL1* (log2FC = −6.85, *p* < 0.0001), and *SLC5A5* (log2FC = −8.98, *p* < 0.0001) were downregulated compared to non‐tumor tissues (Figure 4). Except for *NTN1*, these patterns recapitulated those from the RNA‐seq set, reinforcing their clinical potential.

ROC analysis confirmed good diagnostic performance for all validated genes, with AUC values ranging from 0.78 to 0.85 (Supporting Information S1: Figure S4 and Table S8). *CCNA2* and *SLC5A5* reached the highest AUC (0.85, 95% CI: 0.80–0.89). *CCNA2* displayed the highest sensitivity (91%), while *PSAPL1* showed the highest specificity (86%). In line with its lack of differential expression, *NTN1* failed to show diagnostic value (AUC = 0.45, 95% confidence interval [CI]: 0.38–0.52).

### 3.4 Independent Validation Confirms Robust Discriminatory Performance of the Combined Biomarker Model (Study 1—Validation Phase)

**FIGURE 3:**
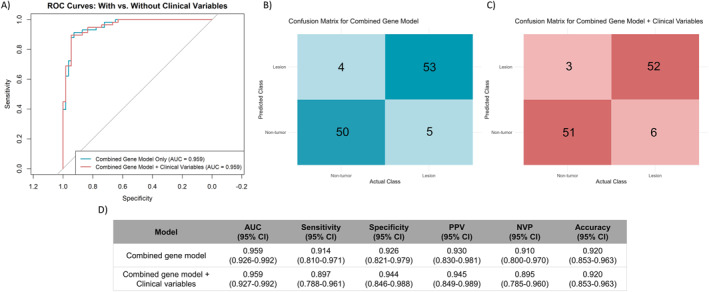
Performance of the multivariate diagnostic models in the validation cohort. (A) Receiver operating characteristic (ROC) curves comparing the combined gene expression model (blue) and the gene model integrated with clinical variables (red). (B) Confusion matrix for the combined gene model alone. (C) Confusion matrix for the combined gene model plus clinical variables. (D) Summary table of diagnostic performance metrics in the validation set with corresponding 95% confidence intervals. AUC: area under the ROC curve, CI: confidence interval, NPV: negative predictive value, PPV: positive predictive value, ROC: receiver operating characteristic.

The six‐gene panel was tested in an independent validation cohort (*n* = 112 samples). The gene‐only logistic regression model demonstrated excellent discrimination (AUC = 0.96, 95% CI: 0.93–0.99, Figure 3A). At the optimal probability threshold (0.72), sensitivity, specificity, PPV, NPV, and accuracy were 0.91, 0.93, 0.93, 0.91 and 0.92, respectively (Figure 3B and 3D). Adding clinical variables did not improve AUC (0.96, 95% CI: 0.93–0.99, DeLong's test *p* = 0.92), though classification metrics varied slightly (Figure 3C and 3D). Sensitivity and NPV fell to 0.90, while specificity and PPV increased to 0.94 and 0.95, respectively, maintaining accuracy.

Calibration and clinical utility measures supported the robustness of both models. The gene‐only model yielded LR+ of 12.34 (95% CI: 4.79–31.78), LR‐of 0.09 (95% CI: 0.04–0.22), and a DOR of 132.5 (95% CI: 33.67–521.64), with a Brier score of 0.082, indicating good model calibration. With clinical variables, higher LR+ (16.14 and 95% CI: 5.35–48.64), a slightly increased LR− (0.11 and 95% CI: 0.05–0.23), and a DOR of 147.3 (95% CI: 34.95–621.06) were observed, with a comparable Brier score of 0.085.

Restricting analysis to HGD and ADC samples produced similarly high AUCs (0.97, data not shown) regardless of clinical variable inclusion (DeLong's *p* = 0.64 and *p* = 0.75).

### 3.5 Exploratory Models Leveraging the Gene Expression Signature Show Potential for Predicting Metachronous Gastric Lesions (Study 2)

To explore whether the tissue‐based gene signature might have relevance for predicting MGL development, we analyzed gene expression in patients with follow‐up data. *HSP90AB1* was significantly upregulated in patients who later developed MGL (log2FC = 1.28, *p* = 0.024, Supporting Information S1: Figure S5), while *HSPD1* exhibited a non‐significant trend toward increased expression (log2FC = 1.21, *p* = 0.094), suggesting potential involvement in lesion recurrence.

Individual ROC curve analyses of each candidate biomarker showed limited predictive ability, with AUC values ranging from 0.49 (*CCNA2,* 95% CI: 0.38–0.59) to 0.61 (*HSP90AB1*, 95% CI: 0.50–0.72, Supporting Information S1: Figure S6). Applying the original diagnostic logistic regression models to MGL prediction resulted in poor discrimination (AUC = 0.48 for the gene‐only model, 0.52 with clinical variables).

**FIGURE 4:**
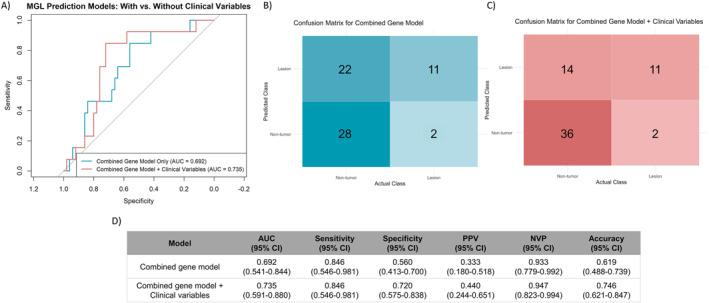
Performance of multivariate diagnostic models for MGL prediction. (A) Receiver operating characteristic (ROC) curves comparing the combined gene expression model (blue) and the gene model integrated with clinical variables (red); (B) Confusion matrix for the combined gene model alone; (C) Confusion matrix for the combined gene model plus clinical variables; (D) Summary table of performance metrics for predicting MGL with corresponding 95% confidence intervals. AUC: area under the ROC curve, CI: confidence interval, NPV: negative predictive value, PPV: positive predictive value, ROC: receiver operating characteristic.

We then trained new multivariable models specifically for MGL and no MGL cases. The gene‐only model achieved moderate discrimination, with an AUC of 0.69 (95% CI: 0.54–0.84, Figure 4A and 4B), sensitivity 0.85 (95% CI: 0.55–0.98), specificity 0.56 (95% CI: 0.41–0.70, Figure 4D), and DOR 7.00 (95% CI: 1.40–34.91). Including age and sex modestly improved performance (AUC = 0.74, 95% CI: 0.59–0.88), specificity (0.72, 95% CI: 0.58–0.84), PPV (0.44, 95% CI: 0.24–0.65), NPV (0.95, 95% CI: 0.82–0.99), and DOR (14.14, 95% CI: 2.78–72.05), while sensitivity remained unchanged (0.85, 95% CI: 0.55–0.98, Figure 4C and 4D). Brier scores were comparable between models (0.167 vs. 0.169), indicating moderate overall calibration and predictive accuracy. Although the integrated model (gene plus clinical variables) showed higher AUC, the difference was not statistically significant (DeLong's test *p* = 0.37), suggesting similar overall discriminative ability. These results indicate the preliminary and exploratory potential of the gene panel for MGL prediction, warranting further validation in larger cohorts.

### 3.6 Salivary *SLC5A5* Is a Potential Non‐Invasive Biomarker for Gastric Lesion Detection (Study 3)

To determine whether biomarkers in tissue could be detected in a non‐invasive matrix, we evaluated candidate gene expression in saliva samples from 102 controls and 95 individuals with gastric lesions. Compared to controls, cases were older and more frequently males. HP infection was more prevalent in controls, while a high proportion of HP‐negative cases may reflect prior eradication or proton pump inhibitor (PPI) use. Lesions were mainly located in the antrum or corpus, with HGD and ADC being the most common histological diagnoses (Table 2).

**TABLE 2:** Clinicopathological features of patients included in the saliva‐based study.

*N* participants/samples	Controls	Cases
	*N* = 102	*N* = 95/99
Age, years		
Mean ± SD	**55.2** ± **12.9**	**66.9** ± **8.1**
Sex, *n* (%)		
Female	**65 (63.7)**	**34 (36.2)**
Male	**37 (36.3)**	**61 (63.8)**
HP status, *n* (%)		
Positive	**35 (34.3)**	**14 (14.1)**
Negative^a^	**60 (58.8)**	**78 (78.8)**
Unknown	**7 (6.9)**	**8 (7.1)**
Localization, *n* (%)		
Antrum		43 (43.4)
Cardia		2 (2.0)
Corpus		25 (25.3)
Incisura angularis		11 (11.1)
Synchronous		11 (11.1)
Corpus‐antrum transition		7 (7.1)
Histology, *n* (%)		
LGD		16 (16.2)
HGD		39 (39.4)
ADC		44 (44.4)

Abbreviations: ADC, adenocarcinoma; HGD, high‐grade dysplasia; HP, *Helicobacter pylori*; LGD, low‐grade dysplasia; MGL, metachronous gastric lesion; SD, standard deviation.

^a^ Eradicated HP cases were classified as negative. Values in **bold** are statistically different between the groups.

**FIGURE 5:**
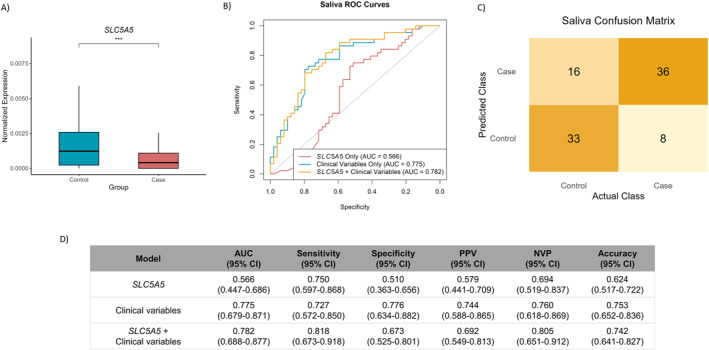
Salivary *SLC5A5* expression and its diagnostic performance for gastric lesion identification. (A) Boxplot showing the expression of salivary *SLC5A5* in cases and controls. (B) Receiver operating characteristic (ROC) curves comparing the *SLC5A5* gene expression alone (red), clinical variables alone (age and sex, blue) and *SLC5A5* integrated with clinical variables (yellow), plotted after cross‐validation. (C) Confusion matrix for the multivariate logistic regression model combining *SLC5A5* and clinical variables. (D) Summary table of performance metrics with corresponding 95% confidence intervals. AUC: area under the ROC curve, CI: confidence interval, NPV: negative predictive value, PPV: positive predictive value, ROC: receiver operating characteristic.

Among the tested genes, all showed detectable expression in saliva, but only *SLC5A5* reproduced dysregulation consistent with tissue results (log2FC = −1.12, *p* < 0.0001, Figure 5A and Supporting Information S1: Figure S7). Expression did not differ between HP‐positive and ‐negative participants (*p* > 0.05, data not shown). Univariate and multivariate analyses confirmed that *SLC5A5* remained significantly associated with gastric lesion risk even after adjusting for age and sex (multivariate odds ratio [OR] = 0.54, 95% CI: 0.29–0.91, *p* = 0.035, Supporting Information S1: Table S9), supporting its independent value.

A logistic regression model incorporating salivary *SLC5A5*, age, and sex was then developed using cross‐validated predictions to avoid overestimation. The combined model reached an AUC of 0.78 (95% CI: 0.69–0.87 vs. AUC = 0.57, 95% CI: 0.48–0.69), Figure 5B, higher than *SLC5A5* alone (AUC = 0.57, 95% CI: 0.48–0.69). At the optimal threshold (0.43), sensitivity was 0.82 (95% CI: 0.67–0.92) and specificity 0.67 (95% CI: 0.53–0.80, Figure 5C and 5D). DOR was 9.28 (95% CI: 3.51–24.51), LR+ of 2.51 (95% CI: 1.64–3.83) and LR‐of 0.27 (95% CI: 0.14–0.52).

Performance was similar to that of clinical variables alone (AUC = 0.775), and DeLong's test indicated no significant difference between models (*p* = 0.66). Still, the combined model showed slightly better overall predictive behavior, including a marginally lower Brier score (0.187 vs. 0.194), higher sensitivity (0.82 vs. 0.73), and improved NPV (0.81 vs. 0.76), desirable in a screening context. These gains are small, but they point to a possible additive contribution of salivary *SLC5A5* within this exploratory setting. Excluding LGD cases did not significantly affect performance (AUC = 0.78; *p* = 0.93).

## 4 Discussion

We report the discovery and validation of a six‐gene expression signature ‐ *ADAMTSL1, CCNA2, HSP90AB1, HSPD1, PSAPL1*, and *SLC5A5 ‐* that accurately discriminates gastric lesions from non‐tumor mucosa in an intermediate‐risk European population. The panel performed consistently across RNA‐seq discovery, qPCR validation, and external TCGA/GTEx datasets.

To develop and validate this signature, we first characterized the transcriptomic alterations associated with gastric carcinogenesis by RNA‐seq using FFPE samples. Through machine learning‐based feature selection, we narrowed down the most promising candidates to a panel of seven genes based on their differential expression patterns and potential clinical relevance. In subsequent validation by RT‐qPCR, six of these genes were consistently dysregulated between gastric lesions and non‐tumor mucosa. Among these, *CCNA2, HSP90AB1*, and *HSPD1* were upregulated, while *ADAMTSL1, PSAPL1*, and *SLC5A5* were downregulated in lesions. These genes are involved in key cellular processes such as cell cycle regulation, stress response, and extracellular matrix remodeling, and some have previously been implicated in GC pathogenesis [25, 26, 27]. The reproducibility of these findings across independent cohorts enhances the robustness of this gene expression panel. In an era increasingly shaped by precision oncology, where risk stratification and early intervention are paramount, the integration of molecular biomarkers into clinical workflows represents a key frontier in GC management [28]. One relevant application is in cases classified as indefinite for dysplasia (IND), where histopathological interpretation remains challenging [17]. Current guidelines recommend repeat high‐definition endoscopy with virtual chromoendoscopy and expert pathology review in these cases, given that up to 29% may harbor invasive carcinoma and nearly 40% are histologically upgraded upon review [17]. Although these cases are uncommon, interobserver variability remains a persistent limitation. In this setting, an objective molecular signature with high diagnostic performance (AUC > 0.95) could provide a valuable adjunct to histology, aiding the interpretation of ambiguous cases by providing complementary information and supporting more consistent, evidence‐based decisions. Beyond diagnosis, exploratory analyses suggested a mild association between *HSP90AB1* expression and subsequent development of MGL. While the integrated model combining gene expression with clinical variables showed a numerically higher AUC than the clinical model alone, this improvement was not statistically significant. These findings should therefore be interpreted as preliminary and hypothesis‐generating, highlighting the potential of the gene panel for MGL prediction but requiring confirmation in larger, independent cohorts. HSP90AB1 protein appears to play a significant oncogenic role in GC by promoting proliferation, invasion, and metastatic potential of gastric epithelial cells [29]. Predicting MGLs remains a critical unmet need, particularly as incidence following endoscopic resection is substantially higher than after surgery (9.5% at 5 years vs. 0.7%) [16].

Our group has recently identified epigenetic alterations, notably *MIR124‐3* and *Nkx‐6.1* hypermethylation, as independent predictors of MGLs in an intermediate‐risk Western population [30]. Building on these findings, the integration of molecular markers, such as this panel, into existing clinical models, such as the FAMISH score, may enhance individualized risk stratification strategies [15]. This is particularly relevant given that current management guidelines recommend managing all primary gastric lesions in the same manner, leading to a monotonous, intensive, and resource‐demanding surveillance [17]. Reliable risk stratification tools could help optimize follow‐up schedules, reducing the clinical, emotional, and financial burdens on patients and healthcare systems, while maintaining safety.

Saliva analysis served as a proof‐of‐concept assessment of whether tissue‐derived markers retained diagnostic relevance in a non‐invasive biofluid. All genes showed detectable signals, but only *SLC5A5* was consistently dysregulated in saliva. This gene encodes the sodium‐iodide symporter (NIS), which is constitutively expressed in gastric epithelium and mediates the uptake of anions such as iodide, nitrate, and thiocyanate [31]. *SLC5A5* downregulation may reduce NIS‐mediated antimicrobial defense in the stomach, through decreased secretion of nitrate and other bactericidal anions into the gastric lumen, creating a microenvironment more permissive to chronic inflammation or bacterial colonization, which could contribute to gastric carcinogenesis [32].

From a clinical perspective, salivary *SLC5A5* should be viewed as a non‐invasive triage biomarker, rather than a standalone diagnostic tool. Its potential utility lies in refining risk stratification and supporting decisions regarding endoscopic evaluation. In a screening‐like context (e.g., individuals undergoing colonoscopy or with non‐specific GI symptoms), a positive result could help prioritize patients for upper GI endoscopy. In surveillance settings, particularly in patients with prior gastric lesions or premalignant conditions, it may help tailor follow‐up by identifying those who could benefit from closer monitoring. These applications remain hypothetical and require prospective validation before clinical implementation.

A multivariable model combining salivary *SLC5A5* with age and sex achieved an AUC of 0.782, with high sensitivity (0.82), an important feature in screening contexts where false negatives must be minimized. *SLC5A5* expression remained independently associated with gastric lesion risk in multivariate analysis, even after adjustment for age and sex, indicating that it contributes beyond demographic factors. While the model based solely on age and sex achieved an AUC of 0.775, nearly identical to that of the combined model (AUC = 0.782), the inclusion of *SLC5A5* led to improvements in sensitivity (0.82 vs. 0.73) and NPV (0.81 vs. 0.76), both critical parameters in screening contexts. Although this difference was not statistically significant (DeLong's *p* = 0.66), the combined model also showed a slightly lower Brier score, indicating better overall calibration and predictive accuracy. Given that some LGD lesions may regress or reflect inflammatory overdiagnosis, we assessed the impact of excluding these cases in both tissue and saliva models. Diagnostic performance remained statistically unchanged, suggesting that inclusion of LGD does not compromise model robustness and that it may still capture clinically relevant alterations, even at very early stages.

**FIGURE 6:**
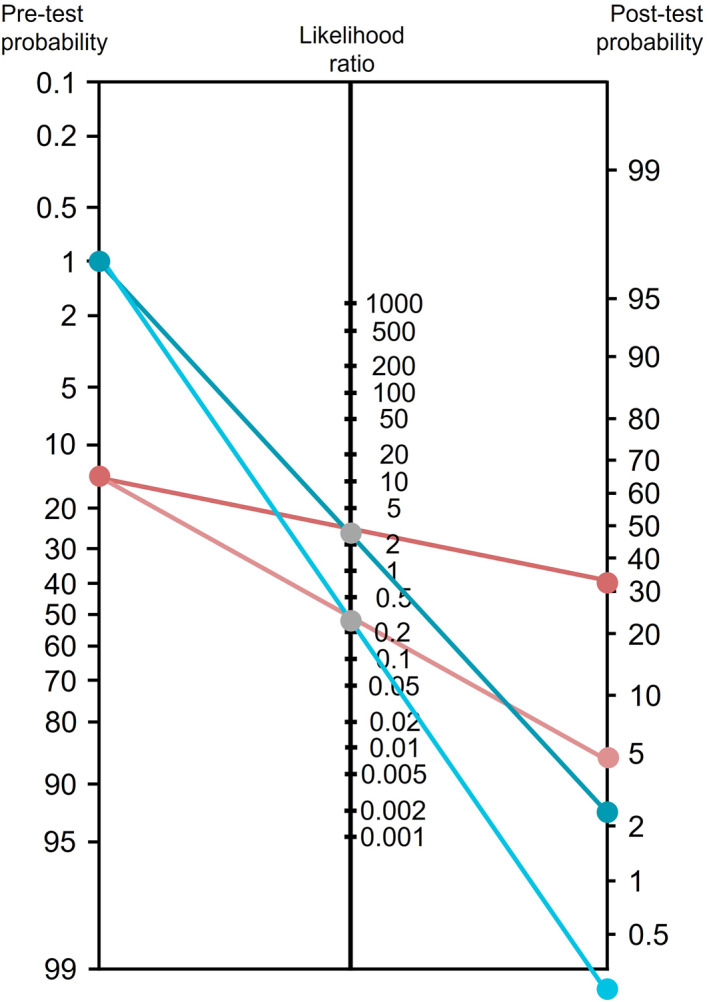
Fagan nomogram illustrating the clinical utility of the salivary *SLC5A5*‐based logistic regression model for gastric lesion detection. Post‐test probabilities were calculated using likelihood ratios derived from the model combining salivary *SLC5A5* expression, age, and sex. Two pre‐test probabilities scenarios were considered: 1% (population‐based screening, in blue) and 15% (prevalence of metachronous gastric lesions, in red).

To contextualize its potential impact, post‐test probabilities were estimated under two scenarios: (1) a screening context, where upper GI endoscopy is offered to individuals undergoing colonoscopy as part of a synergistic approach to digestive tract cancer detection (a strategy shown to be cost‐effective in certain settings and under surveillance scenarios [8, 33]); and (2) a surveillance context, involving patients with a history of gastric lesions undergoing routine follow‐up. In the screening setting, the pre‐test probability of detecting a gastric lesion is estimated to be approximately 1% [34], whereas in the surveillance setting, it rises to ∼15% [15]. A Fagan nomogram (Figure 6) summarizes these results. In the screening context, the model yielded a positive post‐test probability of 2.5% and a negative post‐test probability close to zero. For surveillance, the positive and negative post‐test probabilities were approximately 32% and 5%, respectively. These shifts indicate that salivary assessment may support clinical decision‐making by refining individual risk estimates, potentially guiding the frequency or urgency of follow‐up endoscopy in post‐treatment surveillance protocols.

One candidate gene showed consistent dysregulation in saliva, emphasizing the exploratory nature of this analysis and the potential value of saliva‐first discovery strategies for identifying robust non‐invasive biomarkers. This limited validation aligns with previous work from our group demonstrating that transcriptomic profiles of gastric tissue and saliva differ markedly [35]. Other studies have also developed salivary RNA‐based models for GC detection, although using different approaches. For instance, a Korean study profiled the salivary transcriptome directly to identify a panel of three mRNAs (*SPINK7, PPL*, and *SEMA4B*) and two miRNAs (*miR‐140‐5p* and *miR‐301a*), which yielded an AUC of 0.81, improving to 0.87 with the inclusion of demographic factors [36]. However, this model could not be validated in a U.S.‐based Caucasian cohort, where a new model was derived using the same RNA signature but different combinations, achieving an AUC of 0.78 [37]. These studies highlight the promise of saliva as a diagnostic biofluid but also reinforce the importance of population‐specific biomarker development and the challenge of translating findings across platforms, biofluids, and ethnic groups. Our tissue‐to‐saliva approach demonstrated measurable dysregulation of *SLC5A5* with sensitivity similar to previously reported saliva‐based models in distinct populations, despite differences in discovery strategies and ethnic backgrounds, emphasizing the exploratory nature of these findings.

This study presents several key strengths that reinforce the reliability and translational potential of its findings. A robust identification‐validation framework, further supported by the external validation using independent datasets from TCGA and GTEx, enhances the credibility and generalizability of the proposed gene expression signature. The use of comprehensive statistical approaches, including multivariable logistic regression, ROC analysis, and DeLong's test, ensured a thorough and rigorous evaluation of model performance, yielding diagnostic estimates that are both statistically robust and clinically meaningful. Assessing the same candidates in both tissue and saliva also provides insight into the extent, and limits, of transferring tissue‐derived biomarkers to non‐invasive matrices.

These findings must be interpreted within the biological and methodological constraints of the study. First, transferring tissue‐derived signatures into saliva remains biologically plausible but incompletely understood. Salivary RNA reflects a combination of local and systemic processes, and while some transcripts may reach saliva via extracellular vesicles or other protective mechanisms, the efficiency and consistency of this transfer can be variable. Moreover, the direct transfer of tissue‐derived transcripts into saliva remains speculative, and our findings should be interpreted as proof of concept demonstrating measurable dysregulation of *SLC5A5* in saliva rather than confirming a mechanistic link. Salivary mRNA levels may be influenced by local oral factors, including periodontal disease, variations in salivary flow, and the oral microbiome. Although all samples were collected under standardized fasting conditions and processed to minimize RNA degradation, these variables were not systematically addressed.

Second, the absence of HP status for many control tissue samples limited the ability to adjust for this major confounder [38]. This limitation could not be resolved retrospectively and may have introduced residual confounding in the tissue‐based analyses. In contrast, HP status was available for most saliva participants, and *SLC5A5* expression did not differ between HP‐positive and HP‐negative individuals, indicating that the salivary signal observed here is unlikely to be driven by HP infection.

Third, the application of the diagnostic gene panel to MGL prediction should be interpreted as exploratory. The panel was developed to detect gastric lesions, not MGL, and new models were trained exclusively within the cases (MGL/no MGL) subset.

To advance the clinical applicability of these molecular signatures in GC management, several directions for future research are warranted. Validation in larger, multicenter, and prospective cohorts would be valuable to confirm the performance of both tissue‐ and saliva‐based models across diverse patient populations and clinical settings. In parallel, functional studies exploring the biological roles of the identified genes in early gastric carcinogenesis and MGL development will be important to elucidate their mechanistic relevance and refine their potential clinical utility. Finally, integrating these molecular markers with histological, clinical, and multi‐omics data, such as genomic and proteomic profiles, may enable the development of more comprehensive and precise predictive models. Such integrated approaches have the potential to support individualized risk stratification, improve non‐invasive early detection, and guide personalized management strategies for GC.

## Author Contributions

**C.L.:** conceptualization, methodology, formal analysis, investigation, validation, data curation, visualization, writing – original draft. **A.B.:** methodology, data curation, validation, writing – review and editing. **D.V.:** methodology, data curation, validation, writing – review and editing. **S.P.:** resources, writing – review and editing. **J.C.:** investigation, resources, writing – review and editing. **I.M.d.S.:** investigation, resources, writing – review and editing. **S.A.:** investigation, resources, writing – review and editing. **R.K.‐M.:** investigation, resources, writing – review and editing. **R.M.‐P.:** investigation, resources, writing – review and editing. **D.L.:** investigation, resources, writing – review and editing. **M.D.‐R.:** conceptualization, funding acquisition, project administration, supervision, writing – review and editing. **C.P.:** conceptualization, funding acquisition, project administration, supervision, writing – review and editing.

## Funding

This study was developed in the IPO Porto Research Center (10.54499/UID/00776/2025) and integrated the project AIDA, funded by the European Union and supported by the UK Research and Innovation (101095359 and 10058099, respectively). Views and opinions expressed are however those of the author(s) only and not necessarily reflect those of the European Union or the Health Digital Executive Agency (HaDEA). Neither the European Union nor the granting authority can be held responsible for them. Catarina Lopes (UI/BD/151488/2021) was a research fellowship holder supported by Fundação para a Ciência e Tecnologia (FCT), co‐financed by European Social Funds (ESF) and national funds of MCTES under the Human Strategic Reference Framework (POCH) during the course of this work. Andreia Brandão is supported by the 2021.03835.CEECIND grant by FCT. Carina Pereira holds a Tenure position in the category of Assistant Researcher under the program FCT‐Tenure, with the reference 2023.14811.TENURE.006.

## Ethics Statement

All studies were approved by the Ethics Committee of IPO Porto (CES IPO: 100/024) and conducted according to the Declaration of Helsinki.

## Consent

Written informed consent was obtained from all participants.

## Conflicts of Interest

The authors declare no conflicts of interest.

## Supporting information


Supporting Information S1

## Data Availability

The data for this study have been deposited in the European Nucleotide Archive (ENA) at EMBL‐EBI, a member of the International Nucleotide Sequence Database Collaboration (INSDC), under accession number PRJEB94138. Additional processed data supporting the findings of this study are available from the corresponding author upon reasonable request.
