# Supplementary material for: Molecular Traces of Gastric Cancer in Saliva: From Tissue Signatures to Salivary SLC5A5 as a Potential Biomarker
Source: United European Gastroenterol J. 2026 May 23;14(4):e70221. doi: 10.1002/ueg2.70221 (PMC13239249; doi:10.1002/ueg2.70221)
Supplement: Supplementary file 1 — Supporting Information S1 [file UEG2-14-e70221-s001.docx]

**Supplementary data**

**Methods**

- 1. *RNA extraction*

For RNA-seq analysis, RNA quantification was performed using the Qubit 4 Fluorometer (Invitrogen, Waltham, Massachusetts, USA), and RNA integrity was assessed using the TapeStation system with RNA ScreenTape (Agilent, Santa Clara, CA, USA).

For reverse transcription (RT) real-time polymerase chain reaction (qPCR) and droplet digital (dd)PCR, RNA quantification was conducted with the NanoDrop^TM^ Lite Spectrophotometer (Thermo Fisher Scientific, Waltham, MA, USA), assessing purity by measuring the 260/280 optical density (OD) ratio.

- 1. *RNA-Sequencing and gene expression panel selection*

After rRNA depletion using Illumina Ribo-Zero Plus rRNA Depletion kit (Illumina, San Diego, CA, USA), paired-end libraries were sequenced on the Illumina NovaSeq 6000 platform to ensure high-depth coverage. Following raw sequencing processing, transcript quantification was performed using Salmon (version 1.4.0) in quasi-mapping mode with automatic library type detection and 14 computational threads (18). The reference transcriptome was built using Ensembl release 112 (GRCh38, including both coding and non-coding RNA sequencings. The index was generated from the concatenated cDNA and ncRNA FASTA files. Three control samples were identified as outliers due to the low number of counts and were excluded from the analysis.

Differential expression analysis was carried out using DESeq2 (19). Genes were classified as differentially expressed based on an absolute log-fold change (logFC) threshold greater than 2 (|logFC|>2) and an adjusted P value of less than 0.05 (P-adjust < 0.05). Specifically, logFC>2 indicated upregulated genes, while logFC<-2 indicated downregulated genes.

To identify a promising gene expression panel, the tidymodels framework was employed for preprocessing and feature selection (20). The dataset was divided into training and testing sets using the “rsample” package, allocating 70% of the data for training and 30% for testing. Stratified sampling ensured that the distribution of the target variable was consistent across both sets. The “recipes” package was used to eliminate highly correlated features, setting a threshold of 0.85.

For feature selection, various methods were employed, including random forest (RF), gradient boosting machine (GBM), specifically eXtreme Gradient Boosting (XGBoost), and decision tree (DT). Each model was specified through the “parsnip” package within tidymodels, and key parameters were fine-tuned to optimise performance. A resampling strategy was implemented, dividing the training data into 10 folds and repeating this process five times to ensure robust model performance estimation. A grid search approach was used for hyperparameter tuning, testing up to 10 parameter candidates for each model with the “tune” and “dials” packages. Details on the hyperparameter grids and tuning ranges used for RF, GBM, and DT models are provided in **Table S1**. The top gene expression panel was selected based on the importance scores of the features derived from the best-performing models of all three algorithms, using the “vip” package. The identified gene expression signature was corroborated using publicly available data on The Cancer Genome Atlas (TCGA – stomach adenocarcinoma [STAD] cohort) and Genotype-Tissue Expression (GTEx) portal, filtering for stage I GC patients. A total of 81 stage I GC samples from TCGA, 7 matched normal adjacent tissue (NAT) samples from TCGA and 174 normal tissue samples from GTEx were analysed. The jointly normalised data, in transcripts per million, were obtained using UCSC Xena (<https://xena.ucsc.edu>).

- 1. *Reverse transcription reaction*

Briefly, each 20 µL reaction contained 2.0 µL of 10x RT Buffer, 0.8 µL of 25X dNTP Mix (100 mM), 2.0 µL of 10X RT Random Primers, 1.0 µL of MultiScribe^TM^ Reverse Transcriptase, 4.2 µL of nuclease-free H_2_O and 10 µL of RNA sample.

The reverse transcription (RT) reaction was carried out under the following conditions: 25 ºC for 10 minutes, 37 ºC for 120 minutes, and 85 ºC for 5 minutes. Each reaction included a no-template negative control to ensure specificity.

- 1. *Real-time PCR*

Each 10 µL reaction mix contained 5.0 µL of TaqMan^TM^ Multiplex Master Mix (Applied Biosystems, Waltham, MA, USA), 0.5 µL of each TaqMan^TM^ Gene Expression Assay (Applied Biosystems, Waltham, MA, USA), and 40 µg of cDNA template. The thermal cycling program began with an initial denaturation at 95 ºC for 20 seconds, followed by 45 cycles of 95 ºC for 1 second and 60 ºC for 20 seconds.

Replicates with a standard deviation (SD) exceeding 0.5 were excluded from the analysis. A no-template control was included on each plate to ensure specificity. The endpoint of the real-time PCR was determined by the cycle threshold (C_T_), calculated as the mean of the values obtained from the three independent reactions.

- 1. *Droplet digital PCR*

Each 22 µL of reaction mix contained 5.0 µL of Supermix, 2.0 µL of reverse transcriptase, 1.0 µL of 300 mM DTT, 1 µL of each TaqMan^TM^ Gene Expression Assay, and 50 ng of RNA.

Amplification was performed using the C1000 Touch Thermal Cycler (Bio-rad, Hercules, CA, USA) using the following thermal cycling conditions: reverse transcription at 42 ºC for 60 minutes, enzyme activation at 95 ºC for 10 minutes, 40 cycles of denaturation at 95 ºC for 30 seconds and annealing/extension at 60 ºC for 1 minutes, followed by enzyme deactivation at 98 ºC for 10 minutes and a final hold at 4 ºC for 30 minutes.

- 1. *Data processing and statistical analysis*

Prior to comparative analyses, the distribution of gene expression values was assessed for normality. Depending on distributional properties, either Student’s t-test or the nonparametric Wilcoxon rank-sum test was employed to compare group means or medians, respectively. Differences in continuous variables between groups and sets were evaluated using two-way ANOVA. For categorical variables, chi-square (χ^2^) tests were used when assumptions were met; otherwise, Fisher’s exact test was applied to account low expected frequencies.

The predictive performance of the candidate gene expression signature was evaluated using receiver operating characteristic (ROC) curve analysis with the pROC package (21). The area under the ROC curve (AUC) and corresponding 95% confidence intervals (CI) were calculated to assess the discriminative ability of the models for distinguishing between gastric lesion and control samples, as well as for predicting MGL development. Optimal cut-off points were determined by maximising the Youden Index.

To assess the combined diagnostic potential of the biomarkers, multivariable logistic regression models were constructed using standardised gene expression values as independent variables and sample classification (gastric lesion vs. control) as the outcome. Gene expression data from saliva were standardized (z-score transformed) to perform univariate and multivariate logistic regression analyses. Clinical variables, namely age and sex, known risk factors for GC, were also included as covariates. Predicted probabilities were used to generate multivariable ROC curves and calculate AUC values and optimal thresholds as described. Diagnostic performance metrics, including sensitivity, specificity, positive predictive value (PPV), and negative predictive value (NPV), were estimated along with their 95% CIs using the epiR package. To compare model performances, including the impact of adding clinical variables, DeLong’s test for two correlated ROC curves was used. To further characterise model performance, additional diagnostic accuracy measures were computed, including the positive likelihood ratio (LR+), negative likelihood ratio (LR−), and diagnostic odds ratio (DOR). These were derived from confusion matrix estimates using standard formulae: LR+=sensitivity/(1−specificity), LR−=(1−sensitivity)/specificity, and DOR=LR+/LR−. Model calibration was evaluated using the Brier score, which represents the mean squared difference between predicted probabilities and observed outcomes, with lower values indicating better calibration.

**Supplementary tables**

**Table S1.** Hyperparameter tuning details for machine learning models used in gene expression panel selection

| Model | Hyperparameter | Value |
| --- | --- | --- |
| RF | mtry | 18 |
|  | min_n | 15 |
|  | tress | 1000 (default) |
| GBM | mtry | 20 |
|  | min_n | 4 |
|  | trees | 771 |
|  | tree_depth | 11 |
|  | learn_rate | 0.002 |
|  | loss_reduction | 2.078 x 10^-8^ |
| DT | cost_complexity | 4.232 x 10^-7^ |
|  | tree_depth  min_n | 9  4 |

DT: Decision tree, GBM: gradient boosting machine, RF: random forest

**Table S2.** Clinicopathological characteristics of patients with stage I gastric cancer from the TCGA-STAD cohort

|  | **TCGA-STAD**  **Tumor** |
| --- | --- |
| **Patients** | N = 81 |
| **Age, years** |  |
| Mean ± SD  Median (Min-Max) | 69.14 ± 10.7  71 (41-90) |
| **Gender, n (%)** |  |
| Female | 26 (32.1) |
| Male | 55 (67.9) |
| **Localization, n (%)** |  |
| Antrum/Distal | 22 (27.2) |
| Cardia/Proximal | 13 (16.1) |
| Fundus/Body | 29 (35.8) |
| Gastroesophageal junction | 10 (12.3) |
| Other/NOS | 7 (8.6) |
| **Race, n (%)** |  |
| Asian | 11 (13.6) |
| Black/African American | 3 (3.7) |
| White | 54 (66.7) |
| NA | 13 (16.0) |

NA: Not available, NAT: Normal adjacent tissue, NOS: not otherwise specified, SD: standard deviation, TCGA-STAD: the cancer genome atlas-stomach adenocarcinoma, GTEx: genotype-tissue expression

**Table S3.** Gene expression assay information for target and reference genes, including gene names, IDs and respective dyes, for real-time polymerase chain reaction using formalin-fixed paraffin embedded samples

| Gene | TaqMan^TM^ Assay ID | Dye | Notes |
| --- | --- | --- | --- |
| *ADAMTSL1* | Hs01555850_m1 | ABY | Target gene, panel 1 |
| *CCNA2* | Hs00996788_m1 | FAM | Target gene, panel 1 |
| *HSP90AB1* | Hs03043876_g1 | ABY-PL | Target gene, panel 2 |
| *HSPD1* | Hs01036753_g1 | FAM-PL | Target gene, panel 2 |
| *NTN1* | Hs00924151_m1 | JUN | Target gene, panel 2 |
| *PSAPL1* | Hs04185579_s1 | VIC | Target gene, panel 2 |
| *SLC5A5* | Hs00166567_m1 | JUN-PL | Target gene, panel 1 |
| *TADA2B* | Hs00863614_m1 | VIC | Reference gene, panel 1 |

PL – Primer-limited

**Table S4.** Evaluation of top 10 candidate reference genes for RT-qPCR normalization

| Gene | CV | Rank (Stability) |
| --- | --- | --- |
| *TADA2B* | 0.06892 | 1 |
| *DCLRE1C* | 0.06899 | 2 |
| *SSTR2* | 0.06953 | 3 |
| *CPEB1-AS1* | 0.06991 | 4 |
| *ARSA* | 0.06739 | 5 |
| *RGP1* | 0.07867 | 6 |
| *FMNL1-DT* | 0.07913 | 7 |
| *ZNF844* | 0.08121 | 8 |
| *WBP1L* | 0.08141 | 9 |
| *HOOK2* | 0.08175 | 10 |

CV – Coefficient of variation

**Table S5.** Gene expression assay information for target and reference genes, including gene names, IDs and respective dyes, for droplet digital polymerase chain reaction using saliva samples

| Gene | TaqMan^TM^ Assay ID | Dye | Notes |
| --- | --- | --- | --- |
| *ADAMTSL1* | Hs01555850_m1 | VIC | Target gene, panel 2 |
| *CCNA2* | Hs00996788_m1 | FAM | Target gene, panel 1 |
| *HSP90AB1* | Hs03043876_g1 | Cy5 | Target gene, panel 2 |
| *HSPD1* | Hs01036753_g1 | FAM-PL | Target gene, panel 1 |
| *NTN1* | Hs00924151_m1 | JUN | Target gene, panel 1 |
| *PSAPL1* | Hs04185579_s1 | VIC | Target gene, panel 1 |
| *RPP30* | Hs01124518_m1 | Cy5 | Reference gene, panel 1 |
| *RPS16* | Hs01598518_gH | FAM | Reference gene, panel 2 |
| *SLC5A5* | Hs00166567_m1 | JUN-PL | Target gene, panel 2 |

PL – Primer-limited

**Table S6.** Complete list of packages, corresponding versions and sources used in the analysis (Bioconductor version 3.19)

| Package | Version | Source |
| --- | --- | --- |
| biomaRt | 2.60.0 | Bioconductor |
| caret | 7.0-1 | CRAN |
| clusterProfiler | 4.10.1 | Bioconductor |
| DESeq2 | 1.44.0 | Bioconductor |
| dplyr | 1.1.4 | CRAN |
| epiR | 2.0.84 | CRAN |
| ggplot2 | 3.5.1 | CRAN |
| ggpubr | 0.6.0 | CRAN |
| ggsignif | 0.6.4 | CRAN |
| nortest | 1.0-4 | CRAN |
| org.Hs.eg.db | 3.21.0 | Bioconductor |
| pheatmap | 1.0.12 | CRAN |
| pROC | 1.18.5 | CRAN |
| readr | 2.1.5 | CRAN |
| readxl | 1.4.5 | CRAN |
| reshape2 | 1.4.4 | CRAN |
| stringr | 1.5.1 | CRAN |
| rmarkdown | 2.29 | CRAN |
| rtracklayer | 1.68.0 | Bioconductor |
| tibble | 3.2.1 | CRAN |
| tidymodels | 1.2.0 | CRAN |
| tidyr | 1.3.1 | CRAN |
| tximeta | 1.21.4 | Bioconductor |
| tximport | 1.31.1 | Bioconductor |
| vip | 0.4.1 | CRAN |

CRAN: Comprehensive R archive network

**Table S7.** Estimated coefficients and model parameters for all the logistic regression models.

| **Predictor variable** | **Detection**  **(Genes only)** | **Detection**  **(Genes + Clinical)** | **MGL**  **(Genes only)** | **MGL**  **(Genes + Clinical)** | **Saliva (Gene only)** | **Saliva (Clinical only)** | **Saliva (Gene + Clinical)** |
| --- | --- | --- | --- | --- | --- | --- | --- |
| Intercept | 1.05  (P = 0.005) | 0.99  (P = 0.05) | -1.04  (P = 3.44x10^-5^) | -1.46  (P = 0.0007) | -0.12 (P = 0.58) | -6.18 (P = 7.94x10^-5^) | -5.92 (P = 0.0001) |
| *ADAMTSL1* | 1.54  (P = 0.001) | 1.61  (P = 0.001) | 0.19  (P = 0.45) | 0.07  (P = 0.79) |  |  |  |
| *CCNA2* | -2.12  (P = 9.39x10^-5^) | -2.18  (P =7.62 x10^-5^) | 0.15  (P = 0.62) | 0.04  (P = 0.90) |  |  |  |
| *HSP90AB1* | -0.85  (P = 0.09) | -0.81  (P = 0.11) | -0.45  (P = 0.24) | -0.49  (P = 0.22) |  |  |  |
| *HSPD1* | -0.21  (P = 0.67) | -0.19  (P = 0.70) | -0.31  (P = 0.52) | -0.21  (P = 0.67) |  |  |  |
| *PSAPL1* | 1.04  (P = 0.06) | 1.07  (P = 0.06) | -0.13  (P = 0.70) | -0.12  (P = 0.73) |  |  |  |
| *SLC5A5* | 0.80  (P = 0.15) | 0.88  (P = 0.13) | -0.29  (P = 0.41). | -0.32  (P = 0.37) | -0.51 (0.04) |  | -0.46 (P = 0.10) |
| Scaled Age |  | 0.19  (P = 0.76) |  | 0.44  (P = 0.10) |  | 0.08 (P = 0.0003) | 0.08 (P = 0.0007) |
| Sex (Male) |  | 0.25  (P = 0.42) |  | 0.65  (P = 0.23) |  | 1.54 (P = 0.002) | 1.55 (P = 0.002) |

MGL: Metachronous gastric lesion

**Table S8.** Diagnostic performance of individual tissue-based gene candidates for detecting gastric lesions

| **Gene** | **Sens (95% CI)** | **Spec (95% CI)** | **AUC (95% CI)** |
| --- | --- | --- | --- |
| *ADAMTSL1* | 0.80 (0.71-0.91) | 0.76 (0.63-0.85) | 0.80 (0.75-0.86) |
| *CCNA2* | 0.91 (0.83-0.99) | 0.66 (0.55-0.77) | 0.85 (0.80-0.89) |
| *HSP90AB1* | 0.80 (0.69-0.89) | 0.75 (0.65-0.87) | 0.82 (0.78-0.87) |
| *HSPD1* | 0.80 (0.59-0.89) | 0.69 (0.59-0.88) | 0.80 (0.75-0.85) |
| *NTN1* | 0.67 (0.00-1.00) | 0.37 (0.00-1.00) | 0.45 (0.38-0.52) |
| *PSAPL1* | 0.60 (0.53-0.83) | 0.86 (0.64-0.93) | 0.78 (0.73-0.84) |
| *SLC5A5* | 0.85 (0.77-0.91) | 0.74 (0.66-0.83) | 0.85 (0.80-0.89) |

AUC: area under the ROC curve, CI: confidence interval, Sens: sensitivity, Spec: specificity.

**Table S9.** Univariate and multivariate logistic regression analyses assessing the association between salivary gene expression, age and sex, and gastric lesion risk

OR: Odds ratio. Values **in bold** are statistically significant.

| **Variable** | **Univariate OR (95% CI)** | **Univariate P-value** | **Multivariate OR (95% CI)** | **Multivariate P-value** |
| --- | --- | --- | --- | --- |
| *ADAMTSL1* | **1.501 (1.039-2.245)** | **0.0369** | 1.539 (0.927-2.665) | 0.106 |
| *CCNA2* | 0.964 (0.715-1.30) | 0.801 | - | - |
| *HSP90AB1* | 0.737 (0.538-0.999) | 0.0523 | - | - |
| *HSPD1* | 0.938 (0.692-1.270) | 0.677 | - | - |
| *NTN1* | **0.682 (0.483-0.943)** | **0.0242** | 0.824 (0.479-1.396) | 0.474 |
| *PSAPL1* | 1.072 (0.779-1.481) | 0.669 | - | - |
| *SLC5A5* | **0.463 (0.288-0.692)** | **0.000523** | **0.540 (0.287-0.914)** | **0.0348** |
| *Age* | **1.123 (1.083-1.172)** | **6.28x10^-9^** | **1.093 (1.046-1.150)** | **0.000218** |
| *Sex (Male)* | **2.78 (1.519-5.165)** | **0.00103** | **4.059 (1.608-10.909)** | **0.00388** |

**Supplementary figures**

**
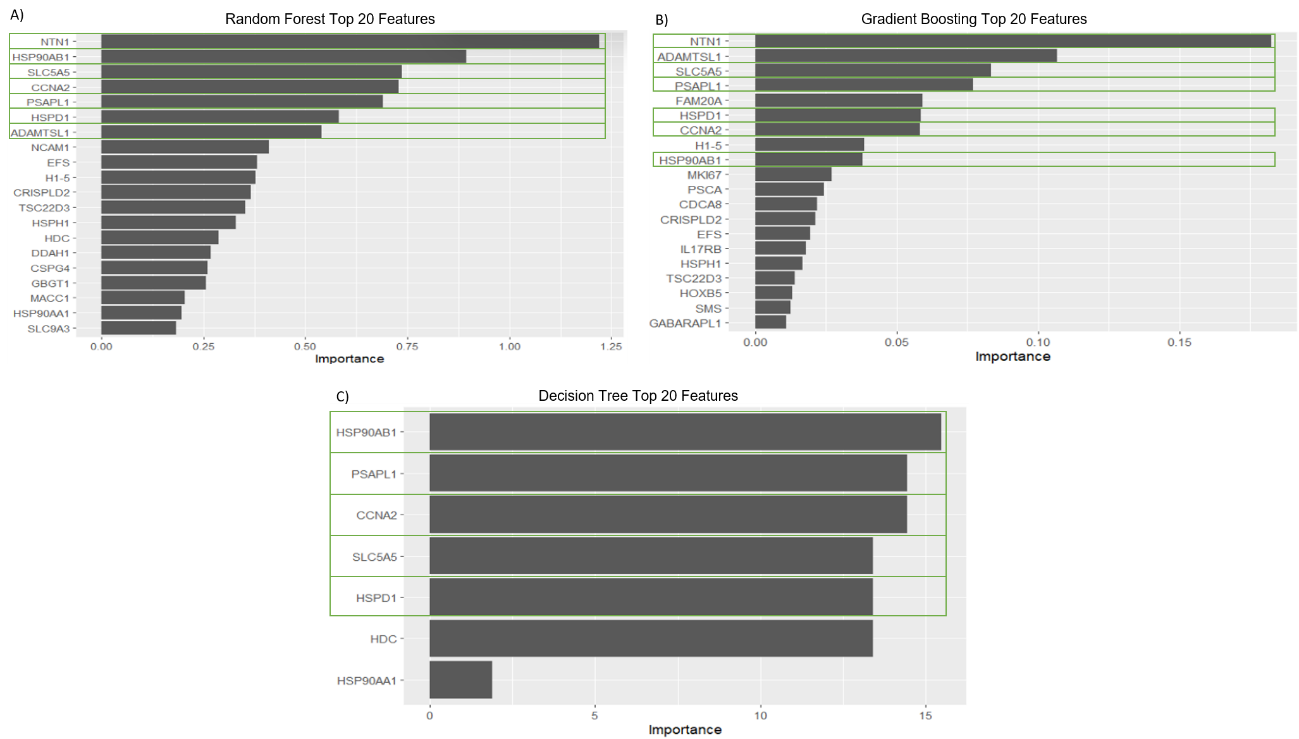
**

**Figure S1.** Feature importance plots for the top 20 features identified by different machine learning models, including A) random forest, B) gradient boosting, and C) decision tree. The selected seven-gene biomarker panel is highlighted within each plot.

**
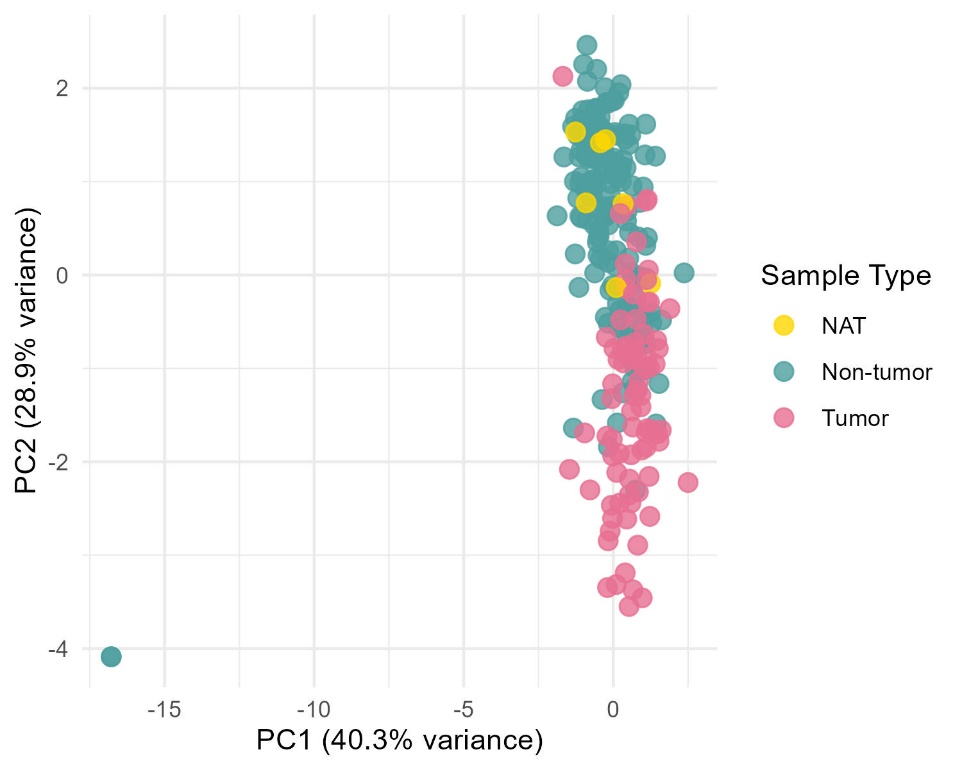
**

**Figure S2.** Principal component analysis (PCA) of stage I gastric cancer and normal tissue samples. PCA was performed to evaluate the expression profiles of the seven-gene panel across stage I gastric cancer samples (The Cancer Genome Atlas-Stomach Adenocarcinoma cohort [TCGA-STAD], normal adjacent tissue (NAT) samples (TCGA-STAD), and non-tumor tissue samples (Genotype-Tissue Expression [GTEx]). The analysis revealed significant overlap between NAT and GTEx non-tumor samples, supporting their combination in subsequent analyses.


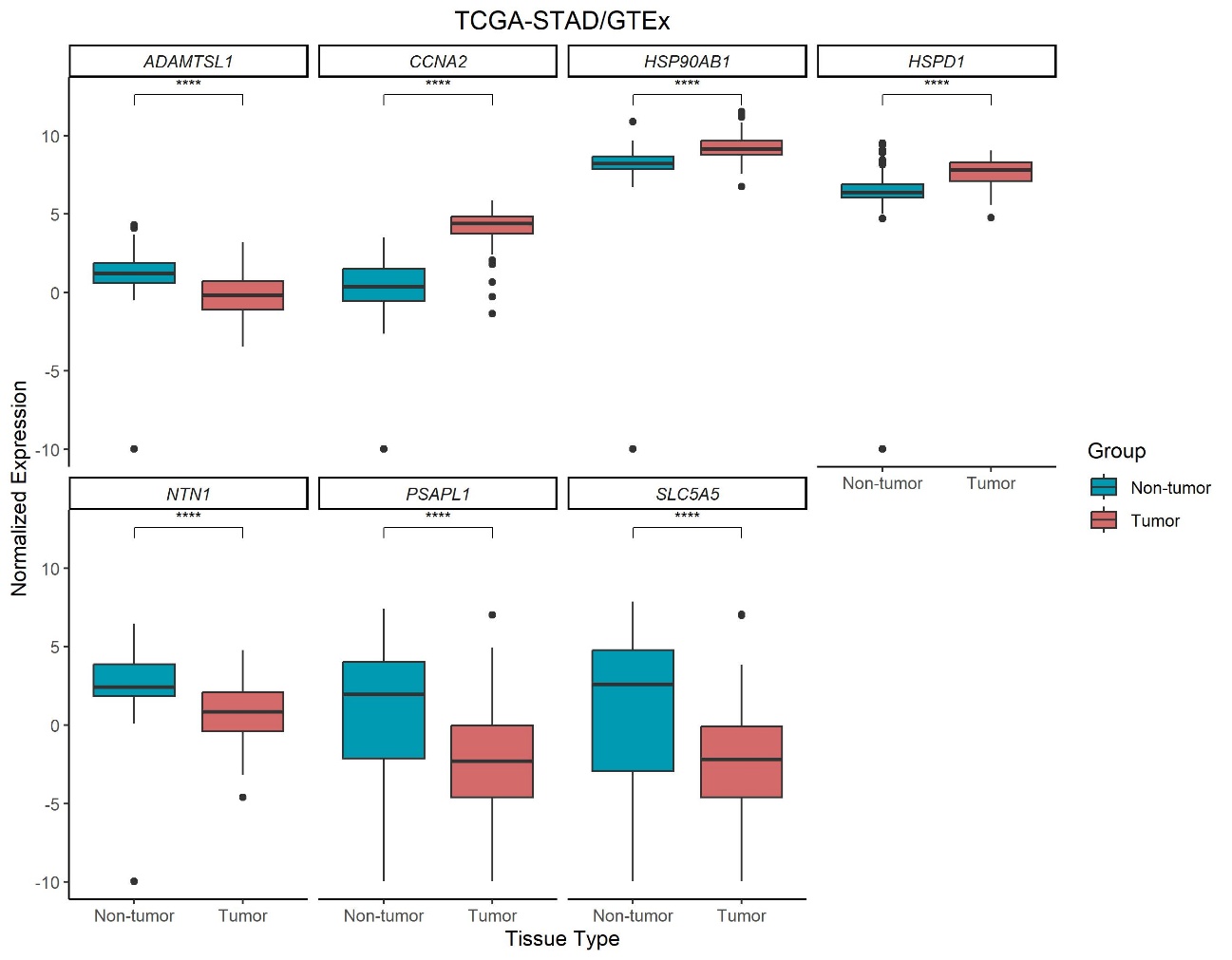


**Figure S3.** Gene expression profile of selected biomarkers in TCGA-STAD/GTEx cohort, comparing non-tumor and tumor samples. Boxplots show the expression levels of the seven genes identified as potential biomarkers for lesions. CCNA2, HSP90AB1 and HSPD1 exhibit significantly higher expression in lesion samples, while ADAMTSL1, NTN1, PSAPL1, and SLC5A5 show lower expression. ****P < 0.0001.

**
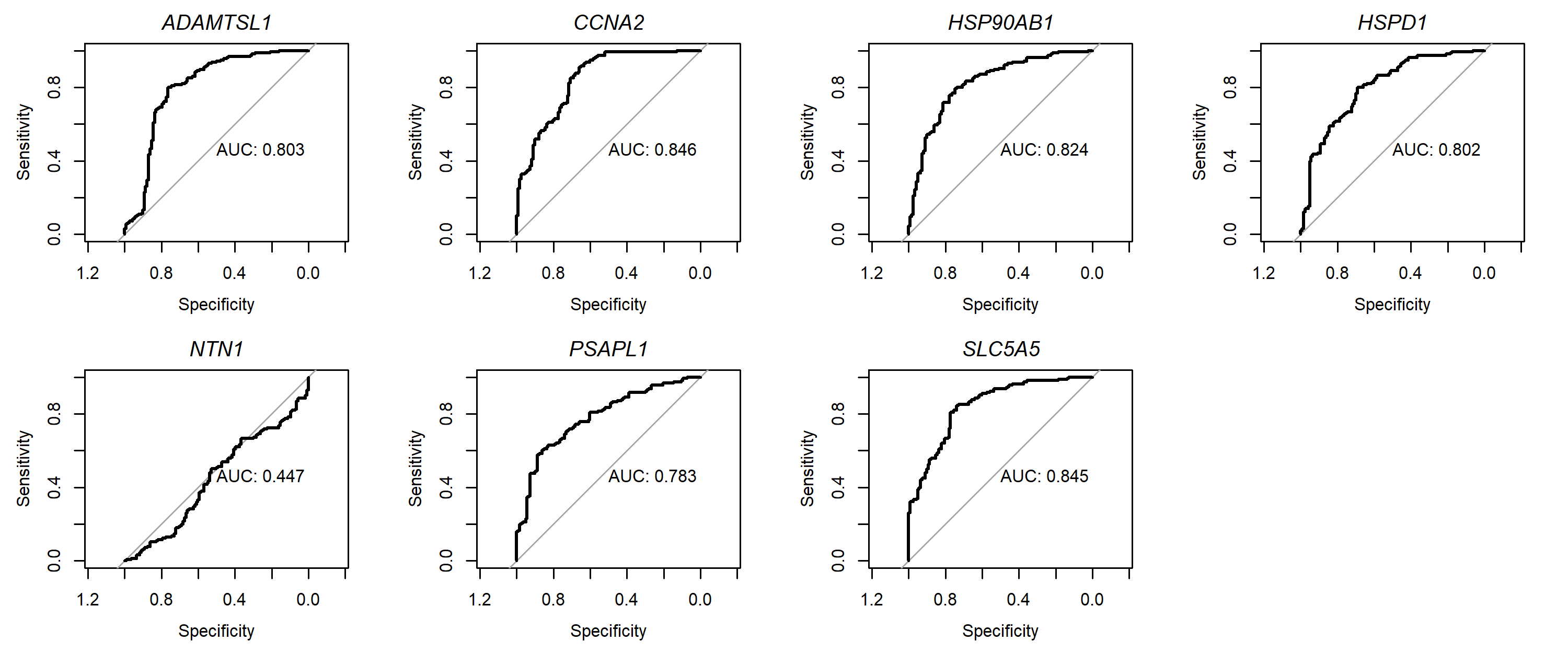
**

**Figure S4.** Receiver operating characteristics (ROC) curves for the seven candidate mRNA biomarkers (*ADAMTSL1, CCNA2, HSP90AB1, HSPD1, NTN1, PSAPL1, SLC5A5*) in discriminating gastric lesion samples from normal mucosa. The area under the ROC curve (AUC) quantifies the discriminatory ability of each biomarker, with values closer to 1.0 indicating higher power.

**
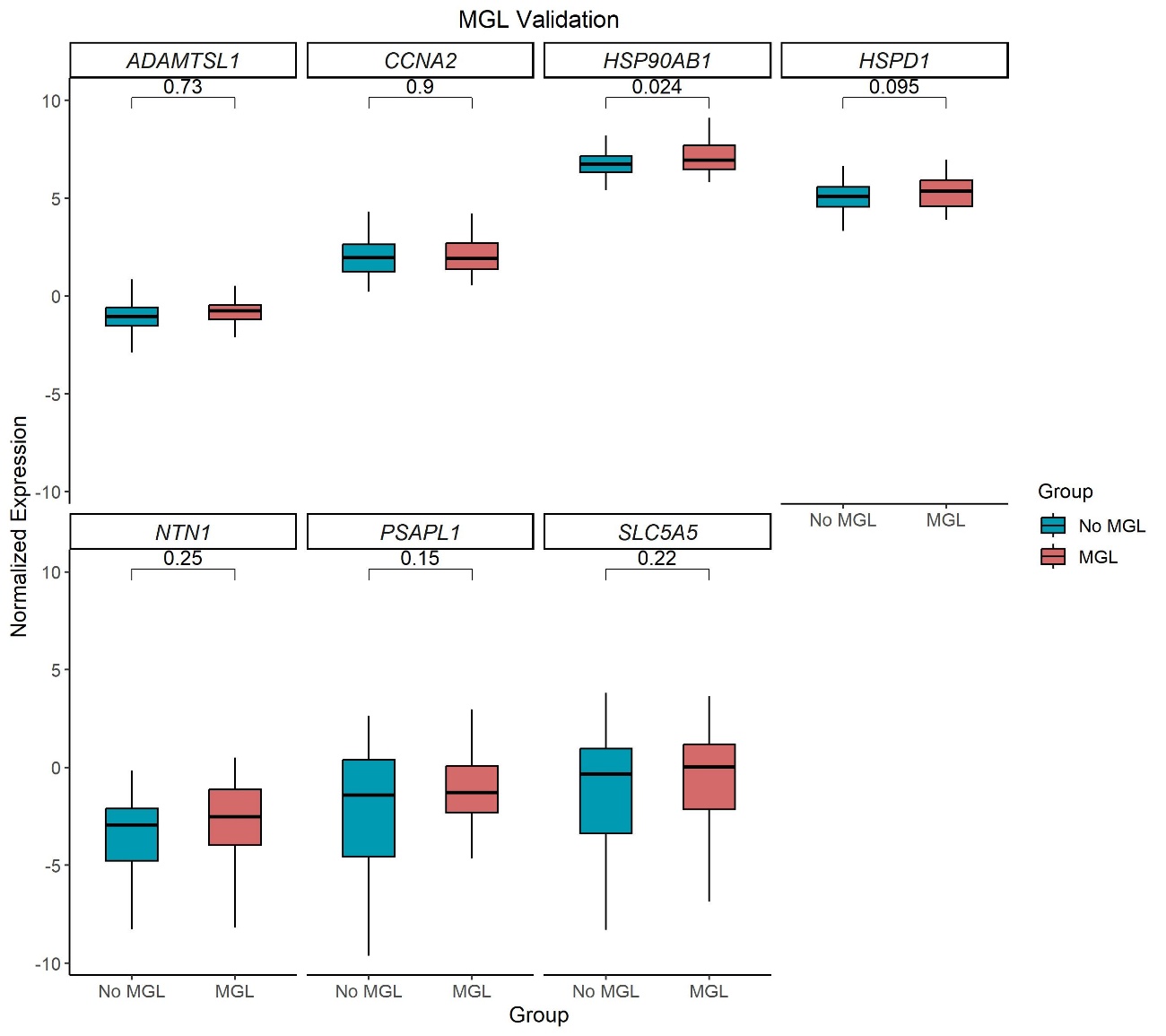
**

**Figure S5.** Gene expression profile of selected biomarkers in stratified cases from the validation cohort according to metachronous gastric lesion (MGL) development. Boxplots illustrate the normalized expression levels (-∆C_T_) of the seven candidate biomarkers stratifying cases from the validation cohort into two groups: MGL development and no MGL development (No MGL) during follow-up. HSP90AB1 exhibited significantly higher expression in MGL samples. MGL: metachronous gastric lesions.

*
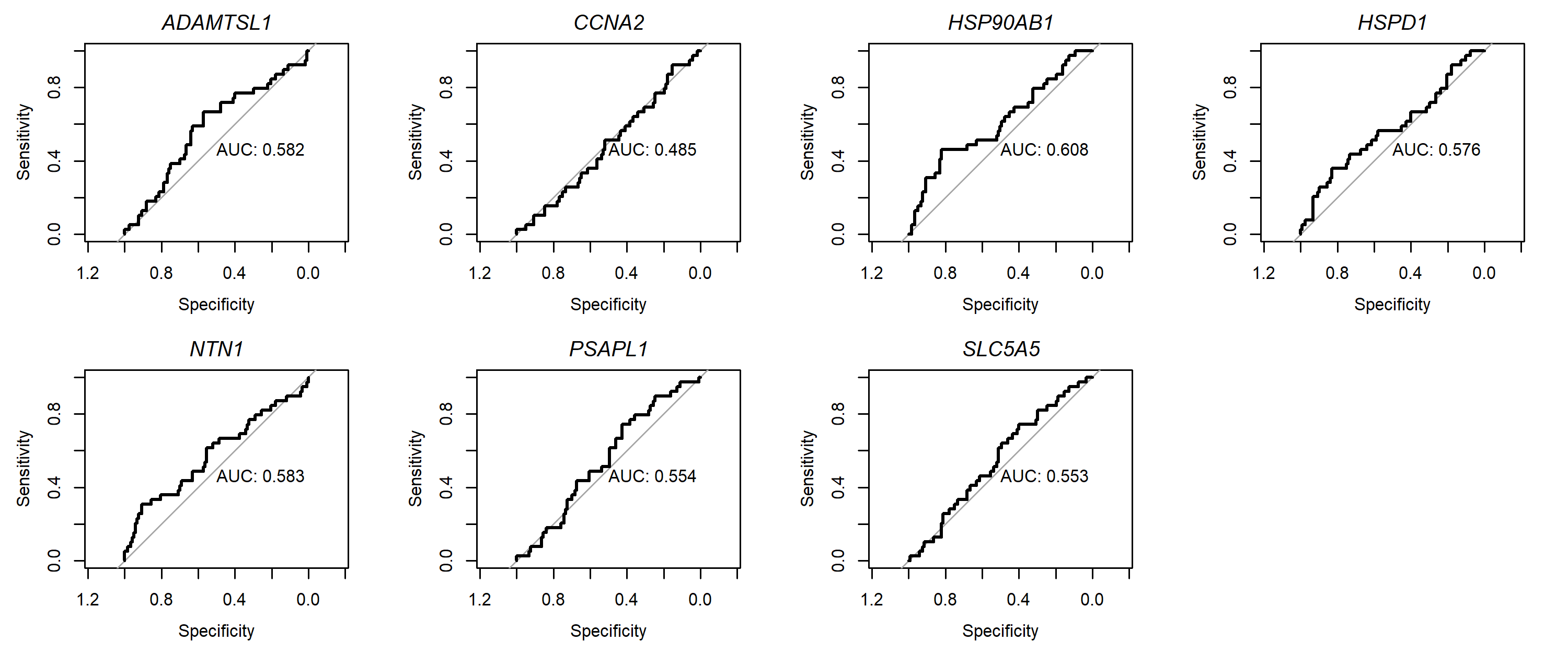
*

**Figure S6.** Receiver operating characteristics (ROC) curves for the seven candidate mRNA biomarkers (*ADAMTSL1, CCNA2, HSP90AB1, HSPD1, NTN1, PSAPL1, SLC5A5*) for predicting the development of metachronous gastric lesions (MGL). The area under the ROC curve (AUC) quantifies the discriminatory ability of each biomarker, with values closer to 1.0 indicating higher power.


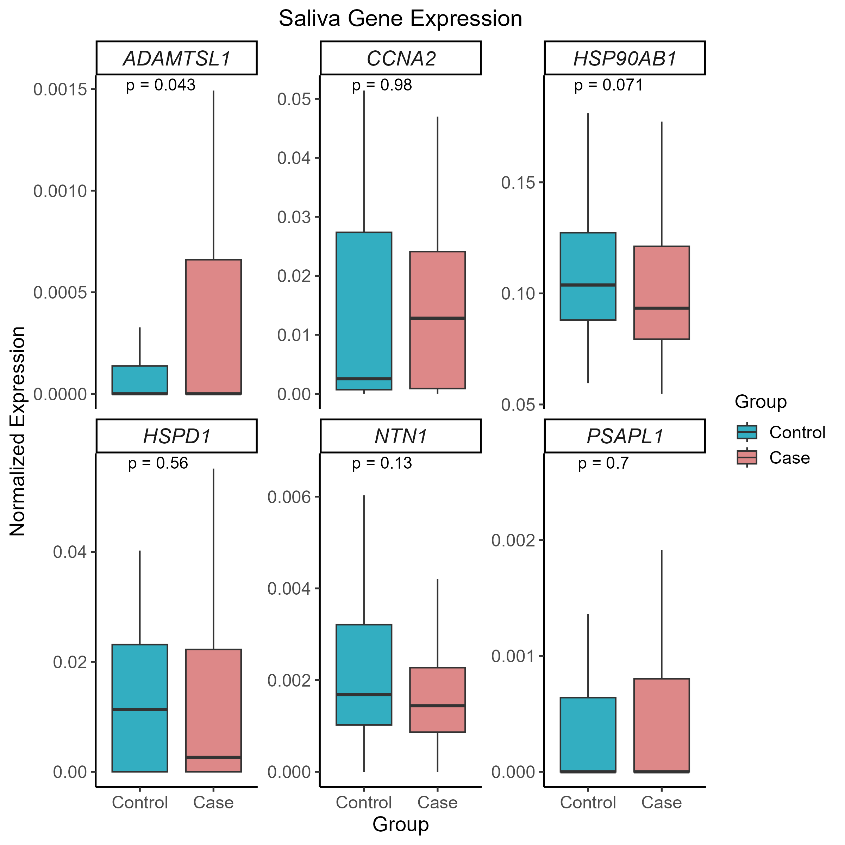


**Figure S7.** Salivary gene expression of candidate biomarkers not retained for further analysis. Boxplots display normalized expression levels (log_10_(expression + 1)) of six candidate genes identified in tissue. Although *ADAMTSL1* showed significant differential expression between cases and controls, the direction of dysregulation was opposite to that observed in tissue, and it was therefore excluded from subsequent analysis.
